# A prospective pilot study of kidney-specific biomarkers to detect acute kidney injury after cytoreduction and hyperthermic intraperitoneal chemotherapy

**DOI:** 10.2478/abm-2024-0034

**Published:** 2024-12-16

**Authors:** Chao-Yu Chen, Ting-Yao Wang, Hung-Yu Chang, Min-Chi Chen, Lan-Yan Yang

**Affiliations:** Department of Obstetrics and Gynecology, Chang Gung Memorial Hospital, Chiayi, Taiwan; Graduate Institute of Clinical Medical Sciences, College of Medicine, Chang Gung University, Taoyuan, Taiwan; Department of Early Childhood Care and Education, Shu-Zen Junior College of Medicine and Management, Kaohsiung, Taiwan; Division of Hematology and Oncology, Department of Internal Medicine, Chang Gung Memorial Hospital, Chiayi, Taiwan; Division of Nephrology, Department of Internal Medicine, Chang Gung Memorial Hospital, Chiayi, Taiwan; Biostatistics Unit of Clinical Trial Center, Chang Gung Memorial Hospital, Linkou, Taiwan; Division of Clinical Trial, Department of Medical Research, Taichung Veterans General Hospital, Taichung, Taiwan

**Keywords:** acute kidney injury, β2 microglobulin, cystatin C, hyperthermic intraperitoneal chemotherapy, neutrophil gelatinase-associated lipocalin

## Abstract

**Background:**

Acute kidney injury (AKI) is a critical morbidity after cytoreduction and hyperthermic intraperitoneal chemotherapy (CRS/HIPEC).

**Objective:**

This study was conducted to investigate the use of kidney-specific biomarkers to evaluate the diagnostic accuracy of post-HIPEC AKI.

**Methods:**

Patients who received CRS/HIPEC were prospectively enrolled in this study. We serially sampled urine neutrophil gelatinase-associated lipocalin (NGAL), serum cystatin C (sCyC), and β2 microglobulin (sβ2-MG) on the day before CRS/HIPEC and then 2 h, 1 d, 2 d, 3 d, and 7 d after CRS/HIPEC. The primary outcome was the occurrence of AKI during the first 7 d. The areas under the receiver operating characteristic curve (AUCs) were calculated to evaluate the detection performance.

**Results:**

A total of 75 patients were eligible, of whom 5 (6.7%) fulfilled the criteria of AKI during the study period (AKI group) and 70 did not (non-AKI group). No significant differences were observed in these biomarkers between the two groups, except for sβ2-MG on day 3 (*P* = 0.025). Regarding changes in biomarker concentrations, the AKI group had a significantly higher concentration range of sCyC on day 3 (*P* = 0.009) and sβ2-MG on day 1 and day 3 (*P* = 0.013 and 0.019).

**Conclusions:**

This is the first prospective study to evaluate the value of kidney-specific biomarkers in patients after CRS/HIPEC. We found that AKI cannot be predicted by simply using the absolute measurements of these biomarkers because of the heterogeneous characteristics of the patients.

Cytoreductive surgery (CRS) after hyperthermic intraperitoneal chemotherapy (HIPEC) is a treatment option for patients with peritoneal carcinomatosis [1]. Acute kidney injury (AKI) is the major morbidity after CRS/HIPEC and can result in prolonged hospitalization, short- and long-term mortality, much higher medical costs, and interference with subsequent treatment plans [2]. The long-term outcome of AKI can lead to chronic kidney disease, progression to end-stage renal disease, and cardiovascular disease [3].

In current clinical care, AKI is diagnosed by monitoring surrogate markers of reduced glomerular filtration rate (GFR), such as an increase in serum creatinine (sCreat) levels and/or a reduction in urine output (UO). These indices are used in the risk, injury, failure, loss, and end-stage kidney (RIFLE) criteria, the acute kidney injury network (AKIN) criteria, and the Kidney Disease Improving Global Outcomes (KDIGO) criteria [4]. In recent years, several studies have shown that novel kidney-specific biomarkers can detect perioperative intrinsic AKI earlier than sCreat [5].

Several studies have discussed the role of these biomarkers in different surgeries. In a meta-analysis of 30 datasets, it was found that neutrophil gelatinase-associated lipocalin (NGAL) in urine and plasma can identify patients at high risk of AKI. The areas under the receiver operating characteristic curve (AUCs) were 0.75 (95% CI, 0.73–0.76) for severe AKI [6]. In patients with traumatic AKI, it was reported that serum cystatin C (sCyC) was significantly higher in the AKI group compared to the healthy group [7]. In contrast, intraoperative levels of serum β2 microglobulin (sβ2-MG) and sCyC in liver transplantation patients have been reported as predictors of postoperative AKI [8].

Few studies have investigated the use of kidney-specific biomarkers in patients undergoing CRS/HIPEC. One study used urine NGAL and kidney injury molecule (KIM)-1 24 h after surgery to monitor 38 patients who were randomized to receive dexmedetomidine. The results showed that these early markers of tubular damage were lower in the dexmedetomidine group but were not directly associated with the severity of AKI [9].

Although previous literature [10] reported that the use of systemic thiosulfate or amifostine before HIPEC might be helpful in preventing AKI, these agents are currently unavailable in Taiwan. Therefore, early predictors of AKI have become essential in the clinic. The aim of this study was to better understand the diagnostic and predictive performance of kidney-specific biomarkers for predicting AKI after CRS/HIPEC.

## Materials and methods

### Study design and study population

This was a prospective observational study conducted at a single institution, Chang Gung Memorial Hospital, Chiayi, Taiwan, from January 2018 to December 2020. Patients who were planning to undergo CRS/HIPEC were recruited consecutively. Exclusion criteria were (1) age <20 or >75 years; (2) preoperative (baseline) creatinine >1.5 dL or estimated GFR <50 mL/min/1.73 m^2^ (to prevent any potential influence on baseline data and mitigate interference that may arise from pre-existing renal functional impairment factors); (3) abnormal liver function with aspartate aminotransferase (AST), alanine aminotransferase (ALT), and bilirubin >3× the normal upper limit; (4) one kidney or previous renal surgery (except ureter stent or percutaneous nephrostomy); or (5) preoperative Eastern Cooperative Oncology Group (ECOG) performance status >2. Based on the average number of patients receiving CRS/HIPEC per year, 150 participants were estimated for this study. This study was approved by the Institutional Review Board of Chang Gung Memorial Hospital (201701400A3), and written informed consent was obtained from all enrolled patients. This study was registered at http://clinicaltrials.gov (NCT 04941625), and the full study protocol is available.

### CRS/HIPEC procedure

All patients underwent a standardized CRS/HIPEC procedure, which has been performed by the multidisciplinary team (MDT) at our hospital since 2015 [2]. The HIPEC procedure was indicated for (1) curative intent of peritoneal metastases from primary or recurrent malignancies with peritoneal metastases, (2) palliation to control ascites, and (3) adjuvant treatment for the prophylaxis of suspicious T4 disease from gastric cancer and colorectal cancer or tumor rupture during surgery. CRS was performed using a midline laparotomy or laparoscopic procedure. After CRS, HIPEC was delivered using the closed method with a PerformerTM HT intraperitoneal hyperthermia system (RanD Biotech). The perfusate was a mixture of normal saline and pentastarch (Haes-Steril, 60 mg/mL, Meda) 10% (3:1) or Dianeal® PD4 peritoneal dialysis solution 1.5% Dextrose (Baxter Healthcare SA). The perfusate was given at a dose of 2 L/m^2^ of the body surface. The chemotherapy was delivered after an intra-abdominal temperature of 41–43 °C had been reached, and the duration of HIPEC was 30–90 min.

### Definition of AKI and outcome measurement

The definition of AKI was based on the RIFLE criteria: R (Risk): increased sCreat × 1.5 or GFR decrease >25% and UO <0.5 mL/kg/h × 6 h. I (injury): sCreat × 2 or GFR >50% and UO <0.5 mL/kg/h × 12 h. F (Failure): increased sCreat × 3 or GFR >75% and UO <0.3 mL/kg/h × 24 h or anuria × 12 h. L (loss): persistent loss of renal function >4 weeks. E (end-stage): end-stage kidney disease >3 months [11].

In our previous retrospective study, we found that the maximum increase in sCreat occurred within 7 d after CRS/HIPEC [2]. Therefore, the primary outcome in this study was defined as the occurrence of AKI, according to the RIFLE criteria during the first 7 d after CRS/HIPEC.

This was an observational study without a blinding procedure. During the study period, the clinical data and the renal outcome by RIFLE criteria might be available to the performers of the renal biomarkers, and the clinical data and renal biomarker results might be available to the assessors of the RIFLE criteria.

### The study protocol and biomarker measurement

All of the patients were discussed at MDT meetings and were recruited preoperatively within 1 month of surgery. Blood samples were obtained during routine phlebotomy at the time of preoperative evaluation or at the induction of anesthesia, and baseline levels of sCyC and sβ2-MG were measured. Baseline urine NGAL was measured after Foley catheter insertion in the operating room. After CRS/HIPEC, levels of sCyC, sβ2-MG, and urine NGAL were measured 2 h postoperatively and then on the morning of day 1 (12–24 h), day 2 (36–48 h), day 3 (60–72 h), and day 7. These samples were measured at the central laboratory of Chang Gung Memorial Hospital. sCyC levels were measured using the latex immunoturbidimetric method with Sekisui reagent (Sekisui Chemical). A normal value of sCyC was defined as 0.57–1.01 mg/L. Urine NGAL was measured using a chemiluminescent microparticle immunoassay (Architect i2000, Abbott) with a normal value defined as <131.7 ng/mL. sβ2-MG was also evaluated using a chemiluminescent immunometric assay (Siemens Healthcare, Llanberis) with a normal value defined as <2,366 ng/mL.

Routine blood data were collected (complete blood cell count and differential count, sCreat, blood urea nitrogen, liver function test, albumin, sodium, potassium, magnesium, calcium, and sugar) preoperatively, 2 h postoperatively, and then on the morning of day 1 (12–24 h), day 3, and day 7.

### Clinical data collection

Data on the patients' characteristics, operative details, postoperative outcomes, and pathology were recorded by the case manager and evaluated by the MDT committee. The data included patient demographics, pre-existing co-morbidities (diabetes, hypertension, and hepatitis), ECOG performance status, cancer type/disease status (primary or recurrence, histological type and grade, and peritoneal carcinomatosis index (PCI) [1]), CRS/HIPEC parameters (chemotherapy regimen, perfusate, cytoreduction time, duration, blood loss, intraoperative blood transfusion, completeness cytoreduction score [1], and temperature), and perioperative fluid status.

### Statistical analysis

Descriptive statistics were used to summarize the demographic and clinical baseline characteristics of the study population. Continuous and categorical variables were presented as median (minimum, maximum) and frequency (percentage), respectively. Patients who developed post-CRS/HIPEC AKI were defined as the “AKI group,” and those who did not were defined as the “non-AKI” group. The range of a biomarker measurement was defined as the difference between the maximum and minimum values during a time frame. The Mann–Whitney *U*-test was used to compare continuous variables between groups, and the chi-square test or Fisher's exact test was performed for categorical variables as appropriate. The AUCs were calculated to evaluate the detection performance among studied biomarkers based on the De Long methods. All tests were two-sided, and a *P*-value <0.05 was considered to be statistically significant. All statistical analyses were performed using SAS version 9.4 (SAS Institute), and the missing data were excluded from the specific analysis. This study was according to the Standards for Reporting of Diagnostic Accuracy Studies (STARD) 2015 guidelines [12].

## Results

### Study population

**Figure 1.**
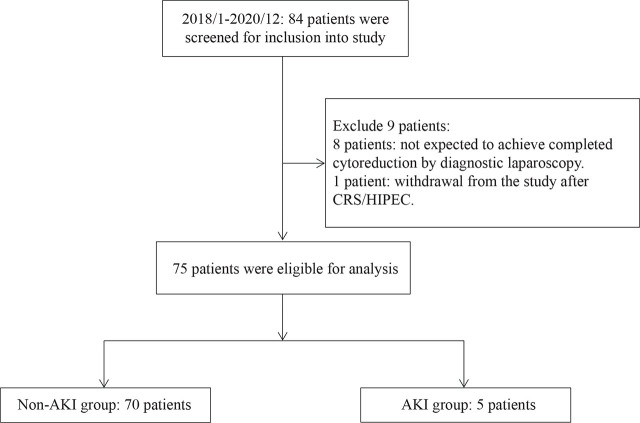
Flowchart of patient enrollment and study population. AKI, acute kidney injury; CRS/HIPEC, cytoreductive surgery with hyperthermic intraperitoneal chemotherapy.

Within the study period, 84 patients were screened preoperatively for enrollment into the study. Eight patients were excluded after the laparoscopic examination revealed that the disease was too extensive, and thus, CRS/HIPEC was canceled. One patient was excluded because they withdrew from the study after CRS/HIPEC. Therefore, a total of 75 patients completed the study and were eligible for analysis. Of them, 5 patients (6.7%, 5/75) fulfilled the criteria of AKI (AKI group) and the other 70 did not (non-AKI group) (**Figure 1**). **Table 1** shows the demographic data, baseline clinical data, and CRS/HIPEC parameters of the study cohort. The median (minimum, maximum) age of all patients was 60 (22–74) years. The most common cancers were gastric cancer (36.0%), ovarian cancer (30.7%), and colorectal cancer (22.7%). The main indication for HIPEC was curative CRS/HIPEC (69.3%); however, 13 patients with gastric cancer underwent adjuvant HIPEC due to T4 disease. Most patients had previously undergone chemotherapy, and only 36.0% of the patients were chemotherapy naïve. There were no significant differences in any of the covariates between the AKI and non-AKI groups. Even though all patients in the AKI group used cisplatin-based HIPEC regimens, the difference between the two groups (100% vs. 57.1%) did not reach statistical significance (*P* = 0.079) **(Table 1)**. The doses of cisplatin in the 5 AKIs were 50 mg/m^2^ (two patients), 75 mg/m^2^ (two patients), and 100 mg/m^2^ (one patient). There were no adverse events from performing the renal biomarkers or the evaluation of RIFLE criteria.

**Table 1.** Demographic and clinical data of the patients enrolled in the analysis (n = 75)

**Characteristics**	**All (n = 75)**	**AKI group (n = 5)**	**Non-AKI group (n = 70)**	***P***
**Demographics**				

Age (years)				0.941
Median (min, max)	60 (22, 74)	55 (48, 68)	60 (22, 74)	
Sex				>0.999
Male	28 (37.3%)	2 (40.0%)	26 (37.1%)	
BMI (kg/m^2^)				0.082
Median (min, max)	24.5 (13, 37)	26.3 (25, 29)	24.2 (13, 37)	

**Baseline clinical data**				

ECOG				>0.999
0–1	71 (94.7%)	5 (100%)	66 (94.3%)	
2	4 (5.3%)	0 (0%)	4 (5.7%)	
Clinical presentation				0.653
Primary	49 (65.3%)	4 (80.0%)	45 (64.3%)	
Recurrence	26 (34.7%)	1 (20.0%)	25 (35.7%)	
Medication before HIPEC				
NSAID	2 (2.7%)	1 (20.0%)	1 (1.4%)	0.130
RAS	4 (5.3%)	1 (20.0%)	3 (4.3%)	0.246
Previous definitive surgery				0.386
None	43 (57.3%)	4 (80.0%)	39 (56.3%)	
Previous systemic therapy				0.093
Never	27 (36.0%)	1 (20.0%)	26 (37.1%)	
First line	27 (36.0%)	4 (80.0%)	23 (32.9%)	
Two lines or more	21 (28.0%)	0 (0%)	21 (30.0%)	
Previous chemotherapy				0.380
Platinum-based	24 (32.0%)	3 (60.0%)	21 (30.0%)	
Other chemotherapy	24 (32.0%)	1 (20.0%)	23 (32.9%)	
Never received chemotherapy	27 (36.0%)	1 (20.0%)	26 (37.1%)	
Preoperative PCI				>0.999
Median (min, max)	6 (0, 39)	6 (0, 25)	6 (0, 39)	
CC score				0.066
0	57 (76.0%)	4 (80.0%)	53 (75.7%)	
1	10 (13.3%)	0 (0%)	10 (14.3%)	
2	2 (2.7%)	1 (20.0%)	1 (1.4%)	
3	6 (8.0%)	0 (0%)	6 (8.6%)	
Enrollment indications				0.744
Adjuvant	16 (21.3%)	1 (20.0%)	15 (21.4%)	
Curative	52 (69.3%)	4 (80.0%)	48 (68.6%)	
Palliation	7 (9.3%)	0 (0%)	7 (10.0%)	
Cancer				0.431
Ovary	23 (30.7%)	3 (60.0%)	20 (28.6%)	
Colorectal	17 (22.7%)	0 (0%)	17 (24.3%)	
Gastric	27 (36.0%)	1 (20.0%)	26 (37.1%)	
Pseudomyxoma peritonei	7 (9.3%)	1 (20.0%)	6 (8.6%)	
Others	1 (1.3%)	0 (0%)	1 (1.4%)	
Comorbidity				
Hypertension	18 (24.0%)	2 (40.0%)	16 (22.9%)	0.588
Diabetes mellitus	15 (20.0%)	2 (40.0%)	13 (18.6%)	0.260
Hepatitis B or C	16 (21.3%)	1 (20.0%)	15 (21.4%)	>0.999

**CRS/HIPEC parameters**				

Surgical method				>0.999
Laparotomy	66 (88.0%)	5 (100%)	61 (87.1%)	
Laparoscopy	9 (12.0%)	0 (0%)	9 (12.9%)	
Blood loss (mL)				0.558
<500	64 (85.3%)	4 (80.0%)	60 (85.7%)	
≥500	11 (14.7%)	1 (20.0%)	10 (14.3%)	
HIPEC regimen				0.079
Cisplatin	45 (60.0%)	5 (100%)	40 (57.1%)	
Non-cisplatin	30 (40.0%)	0 (0%)	30 (42.9%)	
HIPEC perfusate				0.313
Normal saline and pentastarch	53 (70.7%)	5 (100%)	49 (68.6%)	
Peritoneal dialysis solution	22 (29.3%)	0 (0%)	22 (31.4%)	
HIPEC duration (min)				0.582
≤60	58 (77.3%)	5 (100%)	53 (75.7%)	
>60	17 (22.7%)	0 (0%)	17 (24.3%)	

Descriptive statistics are presented as median (min, max) or number (percentage).

AKI, acute kidney injury; BMI, body mass index; CC score, completeness of cytoreduction score; CRS/HIPEC, cytoreductive surgery and hyperthermic intraperitoneal chemotherapy; ECOG, Eastern Cooperative Oncology Group performance status; max: maximum; min: minimum; NSAID, non-steroidal anti-inflammatory drug; PCI, peritoneal cancer index; RAS, renin–angiotensin system antihypertensive medication; SD, standard deviation.

### Serial measurements of kidney-specific biomarkers

The individual trend lines of urine NGAL, sCyC, and sβ2-MG over time and their absolute change values from baseline are shown in **Table 2**. No significant differences or trends in the 3 biomarkers were noted between the AKI and non-AKI groups, except for sβ2-MG on day 3 (*P* = 0.025, **Table 2**).

**Table 2.** Serial measurements of renal markers and electrolytes

**Biomarkers**		**AKI group (N = 5)**	**Non-AKI group (N = 70)**	***P***
sCreat (mg/dL)	Baseline	1.02 (0.69–1.21)	0.78 (0.42–1.24)	0.025^*^
	Post-OP day 1	0.97 (0.70–1.07)	0.74 (0.37–1.34)	0.086
	Post-OP day 3	1.17 (0.55–1.88)	0.62 (0.34–1.25)	0.014^*^
	Post-OP day 7	2.34 (1.49–9.90)	0.67 (0.30–1.49)	0.004^*^
Urine NGAL (ng/mL)	Baseline	10 (10–77.90)	10 (0.30–344.70)	0.570
	Post-OP 2 h	10 (10–32.70)	10.45 (2.90–287.70)	0.416
	Post-OP day 1	10 (10–47.20)	13.45 (3.20–2175.10)	0.254
	Post-OP day 2	10 (10–37.40)	1.47 (2.80–2260.20)	0.478
	Post-OP day 3	10 (10–29.90)	12.25 (2.20–499)	0.696
	Post-OP day 7	16.10 (10.00–20.40)	14.75 (0.80–234.20)	0.562
sCyC(mg/L)	Baseline	1.01 (0.56–1.44)	0.76 (0.40–1.67)	0.066
	Post-OP 2 h	0.69 (0.50–0.76)	0.60 (0.40–1.30)	0.840
	Post-OP day 1	0.71 (0.58–0.88)	0.63 (0.40–1.55)	0.663
	Post-OP day 2	0.83 (0.76–1.24)	0.80 (0.48–1.96)	0.135
	Post-OP day 3	1.17 (0.79–1.27)	0.82 (0.55–1.98)	0.053
	Post-OP day 7	1.38 (0.64–1.63)	0.80 (0.50–1.70)	0.151
Serum β2-microglobulin (ng/mL)	Baseline	1782 (1139–2466)	1449.5 (764–3550)	0.196
	Post-OP 2 h	1381 (1047–1437)	1267.5 (756–3063)	0.857
	Post-OP day 1	1301 (1131–3594)	1279 (694–3574)	0.370
	Post-OP day 2	1414 (1298–1827)	1267 (773–3726)	0.123
	Post-OP day 3	1802 (1452–1880)	1346.50 (865–3336)	0.025^*^
	Post-OP day 7	1649 (1168–2586)	1466 (722–3585)	0.533
Serum sodium (mEq/L)	Baseline	141 (136–144)	140 (126–145)	0.804
	Post-OP day 1	137 (135–137)	137 (128–143)	0.662
	Post-OP day 3	137 (132–142)	137 (126–142)	0.804
	Post-OP day 7	131 (125–135)	135 (118–141)	0.024^*^
Serum potassium (mEq/L)	Baseline	3.70 (3.60–4.10)	4.00 (3.30–4.80)	0.121
	Post-OP day 1	3.80 (3.30–3.90)	3.70 (2.60–4.90)	0.890
	Post-OP day 3	2.90 (2.80–3.90)	3.90 (3.10–5.20)	0.004^*^
	Post-OP day 7	4.30 (2.50–4.90)	3.70 (2.50–5.10)	0.224
Serum magnesium (mEq/L)	Baseline	1.61 (1.51–1.67)	1.64 (1.22–2.21)	0.668
	Post-OP day 1	1.27 (0.91–1.65)	1.42 (0.94–1.71)	0.680
	Post-OP day 3	1.56 (1.22–1.71)	1.57 (1.21–2.05)	0.422
	Post-OP day 7	1.38 (1.16–1.81)	1.47 (0.92–1.88)	0.931
Serum calcium (mEq/L)	Baseline	8.80 (8.70–9.40)	8.85 (7.20–9.70)	0.775
	Post-OP day 1	7.60 (7.00–8.40)	7.90 (6.50–8.70)	0.049
	Post-OP day 3	8.10 (7.80–8.60)	8.30 (7.00–9.40)	0.439
	Post-OP day 7	8.30 (6.90–8.70)	8.40 (7.20–9.70)	0.427
Serum albumin (g/dL)	Baseline	4.45 (4.10–4.70)	4.20 (2.00–4.80)	0.148
	Post-OP day 1	4.10 (3.00–4.50)	3.60 (1.50–4.40)	0.187
	Post-OP day 3	3.70 (3.00–4.20)	3.60 (2.10–4.40)	0.771
	Post-OP day 7	3.40 (2.70–4.00)	3.60 (1.80–4.60)	0.452

Descriptive statistics at each time point are presented as median (min, max).

* *P* < 0.05 was considered to be statistically significant.

AKI, acute kidney injury; NGAL, neutrophil gelatinase-associated lipocalin; Post-OP, post-operative; sCyC, serum cystatin C; sCreat: serum creatinine.

**Figure 2.**
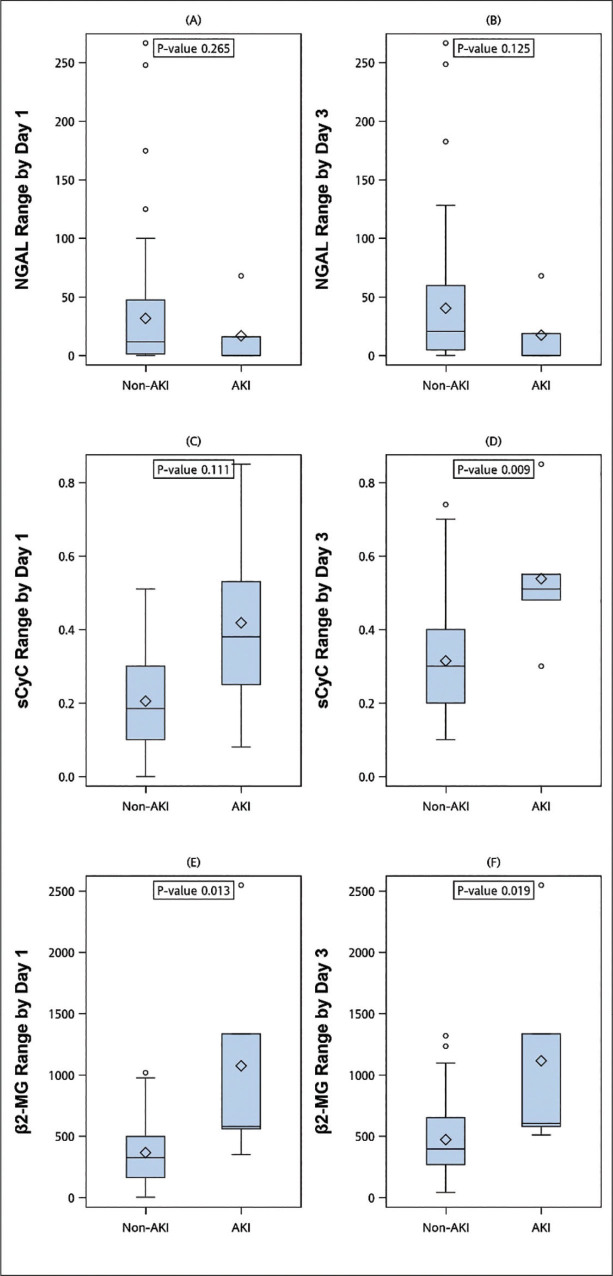
Comparisons of the AKI and non-AKI groups. (**A, C, E**) The ranges of urine NGAL, sCyC, and sβ2-MG between baseline and day 1. (**B, D, F**) The ranges of urine NGAL, sCyC, and sβ2-MG between baseline and day 3. AKI, acute kidney injury; NGAL, neutrophil gelatinase-associated lipocalin; sCyC, serum cystatin C; sβ2-MG, serum β2 microglobulin.

**Figure 3.**
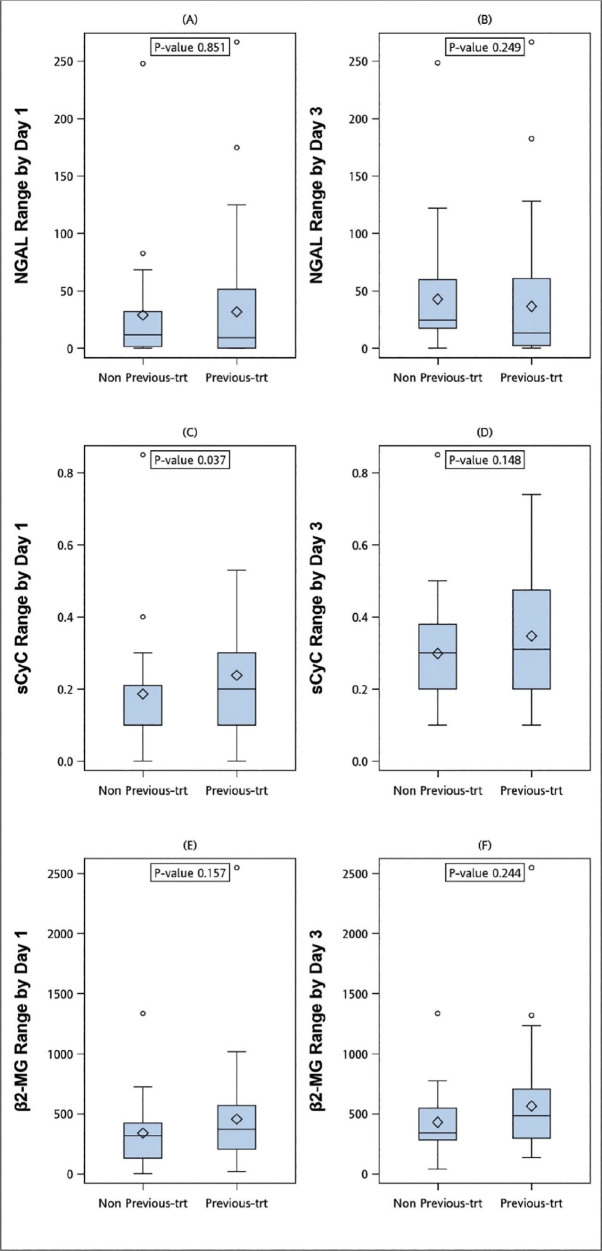
Comparisons of patients without previous chemotherapy treatment (non-previous-trt group) and patients with previous chemotherapy treatment (previous-trt group). (**A, C, E**) The ranges of urine NGAL, sCyC, and sβ2-MG between baseline and day 1. (**B, D, F**) The ranges of urine NGAL, sCyC, and sβ2-MG between baseline and day 3. NGAL, neutrophil gelatinase-associated lipocalin; sCyC, serum cystatin C; sβ2-MG, serum β2 microglobulin.

**Figure 4.**
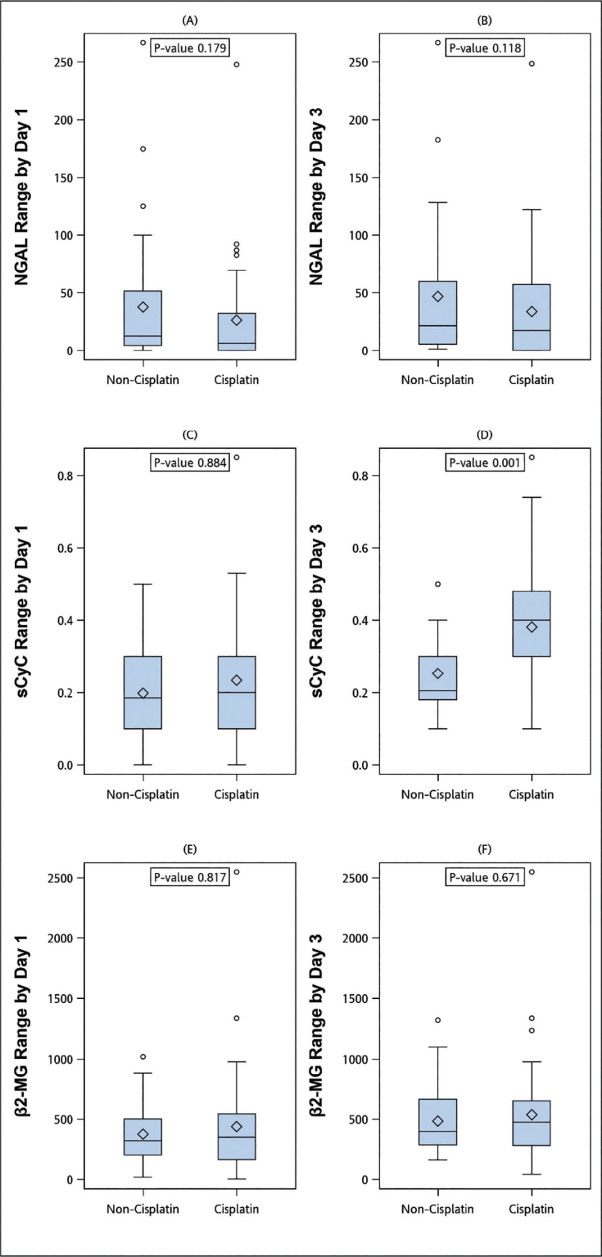
Comparisons of patients who received non-cisplatin HIPEC regimens (non-cisplatin group) and patients who received cisplatin HIPEC regimens (cisplatin group). (**A, C, E**) The ranges of urine NGAL, sCyC, and sβ2-MG between baseline and day 1. (**B, D, F**) The ranges of urine NGAL, sCyC, and sβ2-MG between baseline and day 3. HIPEC, hyperthermic intraperitoneal chemotherapy; NGAL, neutrophil gelatinase-associated lipocalin; sCyC, serum cystatin C; sβ2-MG, serum β2 microglobulin.

The ranges of each studied biomarker concentration from the day before the procedure to day 1 and day 3 are shown in **Figures 2–4**. The AKI group had a significantly higher 3-day range of sCyC concentration (*P* = 0.009, **Figure 2D**) compared with the non-AKI group. For the change in sβ2-MG, the AKI group had a significantly higher range both on day 1 (*P* = 0.013, **Figure 2E**) and day 3 (*P* = 0.019, **Figure 2F**) compared with the non-AKI group.

There was no significant correlation between previous chemotherapy and the occurrence of AKI (*P* = 0.380, **Table 1**); however, the patients who had received chemotherapy before had a significantly higher variation in sCyC on day 1 compared with those who had never received chemotherapy (*P* = 0.037, **Figure 3C**). There were significant differences in the ranges of sCyC on day 3 between the patients who did and did not receive cisplatin (*P* = 0.001) (**Figure 4D**).

### ROC analysis comparing evaluated biomarkers as detecting AK*I*

**Figure 5.**
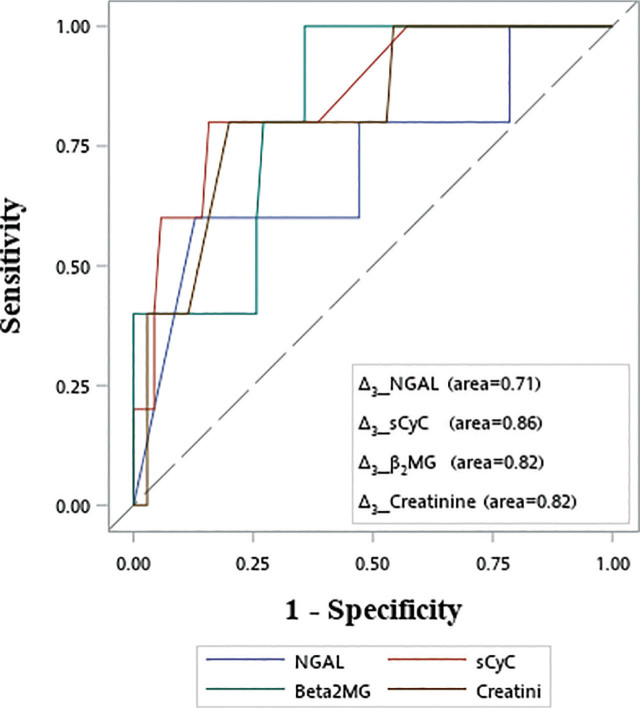
ROC analysis curve showing the range values on day 3 for each study's biomarker to detect AKI. AKI, acute kidney injury; ROC, receiver operating characteristic; sCyC, serum cystatin C; sβ2-MG, serum β2 microglobulin.

Based on the defined normal values of sCyC 1.01 mg/L and sβ2-MG 2366 ng/mL as the cutoffs, the sensitivity was low (20% and 0% of sCyC and sβ2-MG, respectively), while the specificity was high (97% and 93% of sCyC and sβ2-MG, respectively) for detecting AKI. The ROC curves of the range values on day 3 for each study's biomarker to detect AKI are shown in **Figure 5**. The AUC analysis revealed values of 0.71, 0.86, 0.82, and 0.81 for NGAL, sCyC, sβ2-MG, and sCreat, respectively.

## Discussion

In this prospective pilot study, the absolute measurements of urine NGAL, sCyC, and sβ2-MG had limited value in predicting AKI early after CRS/HIPEC. However, we found that the AKI group had significantly higher concentration ranges of sCyC on day 3 and sβ2-MG on day 1 and day 3 compared with the non-AKI group. This result suggests that variations in sCyC and sβ2-MG may be potential predictors of AKI.

In relatively homogeneous populations, kidney-specific biomarkers have been reported to be more useful for assessing early renal outcomes than sCreat, for example, NGAL in patients undergoing cardiac surgery or critical care patients [6]. However, NGAL, a member of the lipocalin family of proteins, may not be a good marker to predict renal outcomes in heterogeneous populations. In the normal kidney, only the distal tubules and collecting ducts stain positive for NGAL [13]. In this study, the distribution of urine NGAL was irregular in both the AKI and non-AKI groups. This may be because the patients with peritoneal carcinomatosis were a heterogeneous population, as they had advanced disease, had possibly received multiple lines of chemotherapy previously, and had comorbidities with underlying diseases or post-treatment effects [14]. Song et al. [9] used NGAL to monitor the effect of dexmedetomidine after CRS/HIPEC and found a lower NGAL concentration in the dexmedetomidine group. However, they did not prove the correlation between NGAL and AKI. Thus, we suggest that clinicians should be cautious when using urine NGAL to predict AKI in CRS/HIPEC patients.

Unlike NGAL, which is secreted by distal tubules and collecting ducts, sCyC and sβ2-MG are metabolized by proximal tubules [5]. CyC is a 13-kDa non-glycosylated cysteine protease inhibitor produced by all nucleated cells and excreted through glomerular filtration [13]. β2-MG is a non-glycosylated protein that is filtered by the glomerulus and is not affected by age or sex [15]. Compared to sCreat, sCyC and sβ2-MG are more specifically related to renal GFR and may be more accurate predictors of AKI [15]. Similar to sCreat, sCyC and sβ2-MG are the “functional” markers of kidney status. However, NGAL is not a functional marker but rather a renal damage marker. Therefore, a rise in sCyC or sβ2-MG could be indicative of a rise in sCreat but not in true renal damage. Ahmad et al. [16] reported that small-to-moderate deteriorations in sCreat or sCyC were not associated with evidence of renal damage, as measured by NGAL and KIM-1. In our study, variations from baseline in sβ2-MG and possibly a trend in sCyC in the AKI group before postoperative day 3 were observed. In summary, changes in sCyC and sβ2-MG over time may be more useful to predict AKI than sCreat measurements, especially when the duration of renal damage is unclear. For example, it may be affected by cisplatin or a large amount of blood loss during the operation, sepsis, complications, or dehydration, which may occur in the postoperative period and cause further renal injury.

All AKIs (n = 5) were treated with cisplatin-based regimens with different dosages in this study. To examine the effect of cisplatin on these markers, we compared the absolute measurement of 3 markers between cisplatin and non-cisplatin groups and found no significant discrepancy (data not shown). However, there were significant differences in the ranges of sCyC values on day 3. The variability of sCyC might be due to the pathophysiology of cisplatin-induced AKI involving proximal tubular injury [17], and the CyC is metabolized completely by the proximal tubules [13]. Further investigation into the application of sCyC in patients with cisplatin-based regimens after CRS/HIPEC is suggested.

The term subclinical kidney damage means damage to the kidney that does not result in a change in sCreat. The novel renal markers could detect this condition [18]. This study enrolled patients with good baseline renal function and potentially well-preserved renal function. Therefore, patients may not have suffered enough tubular damage to induce biomarker elevation or may have appropriate compensatory capability. This is important to measure the AKI diagnosis because short durations of elevation (e.g. <2 d) can reflect prerenal states, rather than true intrinsic kidney damage. Moreover, the occurrence of AKI in patients with negative sCyC, sβ2-MG, or urine NGAL can be attributed to two possible reasons: (1) the etiology of AKI is functional, specifically pre-renal, or (2) the biomarkers may be influenced by confounding factors such as malignancy [19].

Some studies have reported an incidence of AKI after CRS/HIPEC of 11%–40% and also an association between sCreat levels and the development of AKI. However, in our previous study, 12.4% of our patients developed CTCAE grade 1–4 AKI events after CRS/HIPEC [2, 5, 20]. In this cohort, the incidence of AKI was 6.7%, which was lower than the previously reported rate. This difference may be attributed to the enhanced experience in post-CRS/HIPEC care and MDT management, aimed at mitigating risk factors associated with renal injury [2].

There are several strengths to this study. First, to the best of our knowledge, this is the first study to directly evaluate the value of these kidney-specific biomarkers in CRS/HIPEC patients. Although we did not find a significant relationship between these biomarkers and the occurrence of AKI, our results provide a broader understanding of the application of these markers. Second, this was a prospective study, and the biomarkers were compared head-to-head using uniform collection methods and timing, thus avoiding detection bias and missing data. Third, all of the patients in this study were enrolled with the consensus of the MDT, which could minimize performance bias. The major limitation to this study is the relatively small total sample size and the number of patients with AKI (n = 5), which may have resulted in bias and caused the results to be dominated by the non-AKI group. The minor limitation is that the sCreat data were not measured at the same time points as the 3 biomarkers.

## Conclusion

AKI might not be early predicted by simply using the absolute measurements of these kidney-specific biomarkers in patients after CRS/HIPEC because of the heterogeneous characteristics of the patients. However, we observed that variations in sCyC and sβ2-MG have the potential to be predictors. Further studies with more cases to validate these findings are warranted, and a better outcome measurement than creatinine-based criteria should be investigated in future studies.
